# Primary Mammary Angiosarcoma in a Postmenopausal Woman: A Case Report

**DOI:** 10.1002/cnr2.70653

**Published:** 2026-09-09

**Authors:** Tingting Liu, Yueqin Xu, Qingwei Wang, Shiya Yu, Hongming Lin, Zeyi Ye, Zhiqiang Zheng, Changan Yang, Tianzi Hong

**Affiliations:** ^1^ Department of Thyroid and Breast Surgery Jinjiang Municipal Hospital (Shanghai Sixth People's Hospital Fujian) Quanzhou Fujian People's Republic of China; ^2^ Department of General Surgery Fuzong Clinical Medical College of Fujian Medical University, 900th Hospital of the Joint Logistics Support Force Fuzhou Fujian People's Republic of China; ^3^ Department of Thyroid and Breast Surgery Shishi General Hospital Quanzhou Fujian People's Republic of China

**Keywords:** breast mass, case report, middle‐aged female, primary breast angiosarcoma, rare breast malignancy

## Abstract

**Background:**

Primary breast angiosarcoma (PAS) is a rare aggressive mesenchymal breast tumor, which predominantly affects young premenopausal women and is prone to misdiagnosis due to nonspecific clinical and imaging features. Surgical resection is the main treatment, while the efficacy of adjuvant chemoradiotherapy remains unclear. Targeted therapy represents a promising novel therapeutic direction.

**Case:**

A 51‐year‐old postmenopausal woman presented with a rapidly enlarging left breast mass for over 3 months. Physical examination showed a 4 cm × 3 cm × 2 cm mass with a smooth surface, moderate mobility and no cutaneous changes. The lesion was radically resected via two‐stage surgery, and the patient was diagnosed with stage I (T1N0M0) primary left breast angiosarcoma. The patient received sequential adjuvant chemotherapy with doxorubicin plus cyclophosphamide followed by paclitaxel. No recurrence or metastasis occurred during more than 5 years of follow‐up.

**Conclusion:**

Different from conventional PAS predominantly occurring in young women, this atypical postmenopausal PAS has high concealment and diagnostic difficulty. This study summarizes the clinical characteristics and diagnostic pitfalls of this rare case, supplements the clinical and prognostic data of postmenopausal PAS, and helps improve clinicians' recognition of atypical PAS to avoid delayed diagnosis and treatment.

**Abbreviations**
BIRADS: breast imaging reporting and data system
CA—125: carbohydrate antigen 125
CA—153: carbohydrate antigen 15–3
CA—199: carbohydrate antigen 199
CDUS: color Doppler ultrasound
CEA: carcinoembryonic antigen
CT: computed tomography
ECG: electrocardiogram
ERG: ETS—related gene
FLI—1: friend leukemia integration 1 transcription factor
FVII: factor VII‐related antigen
MDT: multidisciplinary team
MG: mammography
MRI: magnetic resonance imaging
NGS: next‐generation sequencing
OS: overall survival
PAS: primary breast angiosarcoma
PIK3C‐α: phosphatidylinositol‐3‐kinase catalytic alpha subunit
RFS: the recurrence‐free survival
SAS: secondary angiosarcoma of the breast
UEA—1: ulex europaeus agglutinin I

## 1 Introduction

Primary breast angiosarcoma, a rare malignant tumor originating from endothelial cells lining the blood vessel, accounts for approximately 0.04% of primary malignant breast tumors [1]. It predominantly occurs in young women aged 20–40 years, with atypical clinical manifestations characterized by a rapidly growing breast mass, and is usually not associated with a history of radiation or chemical toxin exposure [2]. This case report discusses a postmenopausal middle‐aged female patient with PAS who presented with the chief complaint of “finding a left breast mass for more than 6 months.” After completing relevant clinical examinations and preparations, the patient underwent two breast surgeries in our hospital, and the final pathological diagnosis was PAS. This report will summarize the experience and lessons learned from the patient's medical consultation process, and discuss the diagnosis and treatment of such diseases, so as to improve the differentiation and attention to this disease.

## 2 Case Report

### 2.1 Patient History

A 51‐year‐old female patient was admitted to our hospital with the chief complaint of “finding a left breast mass for more than 6 months.” She first detected the left breast mass in September 2019, with a size of approximately 2 cm × 1 cm × 1 cm. The mass had good mobility, without local tenderness, skin changes, nipple discharge, or other symptoms, so she did not pay attention to it or receive any diagnosis and treatment. In November 2019, she visited another hospital and underwent mammography (MG) and breast color Doppler ultrasound (CDUS) examinations. The size of the mass showed no significant change compared with the previous one, and it was considered a benign lesion, with a suggestion of follow‐up. Over the subsequent 3 months, she felt a significant enlargement of the mass, which was approximately 4 cm × 3 cm × 2 cm, occasionally accompanied by pain, and other symptoms remained unchanged. Therefore, she was admitted to our hospital in March 2020. Physical examination on admission: The skin of both breasts was smooth, without redness, swelling, or orange peel‐like changes, and the skin temperature was normal. A mass could be palpated in the left breast, measuring approximately 4 cm × 3 cm × 2 cm, with a smooth surface, moderate texture, relatively clear boundary, good mobility, no fluctuation, and no local skin ulceration. No mass was palpable in the right breast. There was no nipple retraction or discharge in either breast. No obvious enlarged lymph nodes were palpable in the bilateral axillae and superficial regions. Past medical history: She underwent “laparoscopic total hysterectomy” in our hospital in March 2019, with a smooth operation and good postoperative recovery. She denied a family history of breast cancer‐related tumors, as well as a history of exposure to radioactive substances and chemical toxins.

### 2.2 Laboratory and Imaging Examinations

**FIGURE 1:**
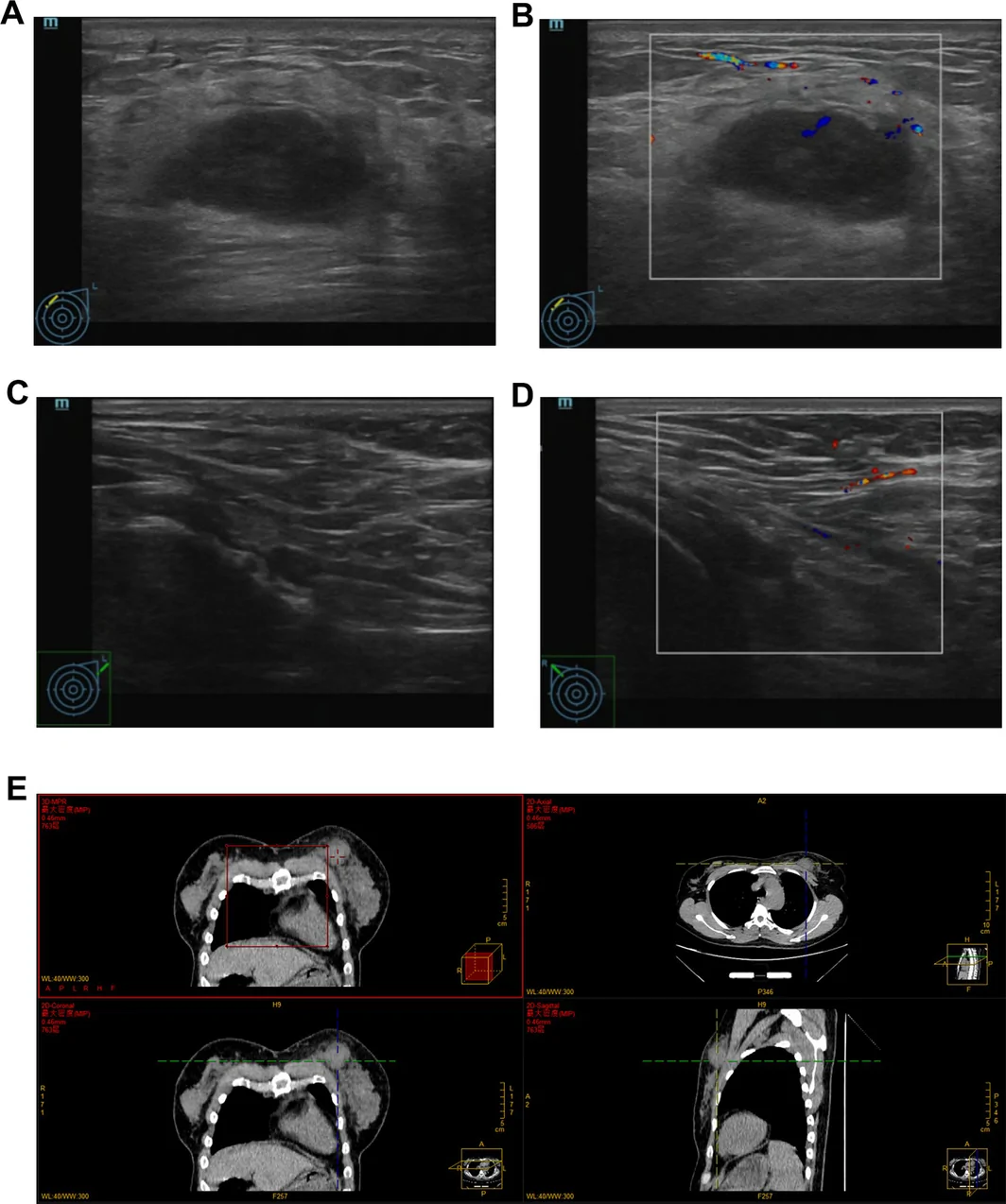
Initial breast color Doppler ultrasound (CDUS) and chest CT images obtained on March 6, 2020. (A–D) Breast CDUS images: Left breast mass (A), vascular blood flow signal of the mass (B), and bilateral axillary CDUS with no enlarged lymph nodes (C–D). (E) Multiplanar localized chest CT image of the left breast mass.

On November 20, 2019, the patient underwent MG in another hospital, which showed a mass in the posterior space of the left breast with benign—leaning signs, classified as Breast Imaging Reporting and Data System (BIRADS) category III. Simultaneously, breast CDUS revealed a hypoechoic nodule in the left breast, measuring approximately 1.8 cm × 1.0 cm × 0.8 cm, categorized as BIRADS category III, and follow—up observation was recommended. Subsequently, the breast mass enlarged significantly, so the patient visited the outpatient department of our hospital on March 6, 2020. Breast CDUS showed a solid space—occupying lesion in the left breast (BIRADS category 4a), with a size of approximately 3.54 cm × 2.39 cm × 1.45 cm, located at the 11 o'clock position, about 6.4 cm away from the nipple. The lesion had an irregular shape, an aspect ratio of less than 1, lobulated margins, and internal hypoechogenicity, without obvious calcification shadows. There was no significant change in posterior echo, and strip—like blood flow signals were observed inside, from which low—resistance arterial blood flow could be detected. No obvious space—occupying lesions were found in the right breast, no obvious abnormal blood flow signals were observed in the glandular tissue, which was consistent with the sonographic changes of bilateral breast hyperplasia. No obvious enlarged lymph node echoes were found in the bilateral axillae (Figure 1A–D). Chest computed tomography (CT) showed a mound—shaped mass shadow in the upper inner quadrant of the left breast, with a size of approximately 4.3 cm × 2.4 cm, and the boundary was unclear on plain scan (Figure 1E). CDUS of the digestive and urinary systems showed changes after total hysterectomy, and no other obvious abnormalities were found; electrocardiogram (ECG) showed no obvious abnormalities.

Laboratory examinations, including blood routine, liver and kidney function, electrolyte tests, coagulation function, infectious disease screening, and urine routine, showed no obvious abnormalities. The levels of relevant tumor markers were all within the low range: carcinoembryonic antigen (CEA): 3.25 ng/mL (reference value: 0–5 ng/mL), carbohydrate antigen 15–3 (CA—153): 6 u/mL (reference value: 0–20 u/mL), carbohydrate antigen 125 (CA—125): 13 u/mL (reference value: 0–22 u/mL), and carbohydrate antigen 199 (CA—199): 8.19 u/mL (reference value: 0–30 u/mL).

Based on the patient's medical history and physical findings, mammography assigned BI‐RADS 3 classification, whereas concomitant ultrasonography upgraded the left breast lesion from BI‐RADS 3 to BI‐RADS 4A. However, ultrasound interpretation is prone to inter‐operator subjective bias. After comprehensive integration of clinical manifestations and dual imaging results, the lesion was highly suggestive of benign fibroadenoma. Per the 5th edition ACR BI‐RADS criteria, NCCN breast diagnostic guideline (V1.2024) and CSBrS 2021 fibroadenoma clinical guideline, the patient's combined clinical and imaging features failed to satisfy formal indications for contrast‐enhanced breast MRI, whole‐body bone scintigraphy or percutaneous core needle biopsy [3, 4, 5].

### 2.3 Clinical Course and Pathological Results

On March 6, 2020, the patient visited the outpatient department of our hospital, where she underwent breast CDUS, chest CT, and relevant hematological examinations. Combined with the patient's past medical history and symptoms, we made a preliminary diagnosis of “left breast space‐occupying lesion: highly likely benign fibroadenoma” and recommended elective hospitalization for surgical resection. The patient was admitted to the Department of Thyroid and Breast Surgery of our hospital on March 10, 2020. After completing relevant preoperative preparations, she underwent “left breast mass resection” under general anesthesia on March 11, 2020. Intraoperative frozen section pathology suggested: (left breast mass) spindle cell tumor, tending to be a vascular‐derived tumor, pending further diagnosis by immunohistochemistry. Since we had rarely encountered such cases previously, after consulting with the patient's family, it was decided to temporarily terminate the surgery, and subsequent treatment would be performed after the diagnosis was confirmed by further pathological results.

**FIGURE 2:**
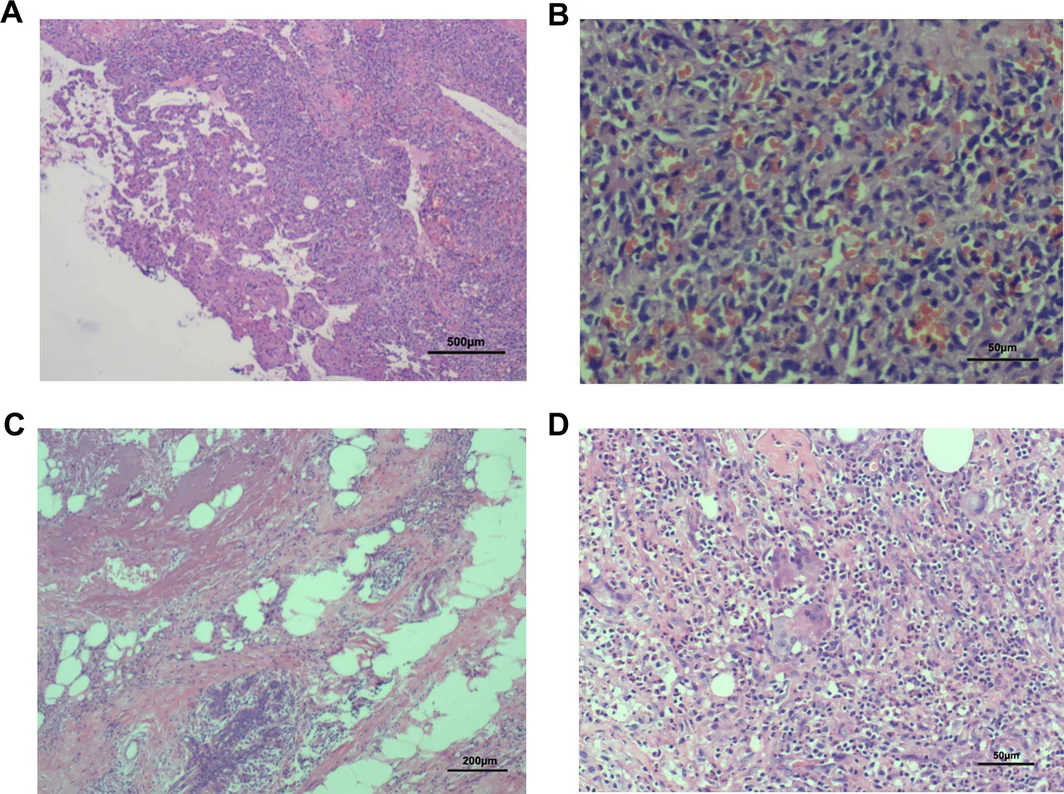
HE staining of two surgical specimens. (A–B) Histological features of the first resected left breast mass on March 11, 2020 (A: ×40; B: ×400). (C–D) Histological features of residual left breast tissue resected on March 23, 2020 (C: ×100; D: ×400).

On March 17, 2020, the postoperative pathological results were obtained: an irregular tissue specimen was submitted, and a nodule was observed upon incision, with a size of approximately 3.5 cm × 2.5 cm × 1.8 cm. The submitted tissue showed a large number of anastomosing slit‐like vascular lumens, which infiltrated and grew interspersed within lobules and surrounding adipose tissue, and destroyed the lobular structure. Red blood cells were visible in the lumens, and the lumen walls were lined with a single layer of endothelial cells with mild nuclear pleomorphism and occasional mitotic figures. Immunohistochemical results: CD34 (+), CD31 (+), FLT‐1 (+), Vimentin (+), SMA (+), EMA (−), CKpan (−), H‐Caldesmon (−), Ki67 (+, 25%), ER (−), PR (−), GCDFP‐15 (−). Combined with immunohistochemistry, the lesion was suggestive of low‐grade angiosarcoma (Figure 2A,B), and pathological section consultation was recommended.

Meanwhile, the patient's pathological sections were sent to a higher‐level hospital for consultation. On March 20, 2020, the consultation result was returned: the submitted breast tissue showed part of the tumor consisted of a large number of anastomosing slit‐like lacunae covered with relatively mild epithelial morphology, while the other part was solid with dense arrangement of tumor cells, obvious pleomorphism, visible mitotic figures, and extensive hemorrhage and necrosis. The tumor cells surrounded the residual breast tissue. Combined with the immunohistochemical results, the diagnosis was consistent with angiosarcoma, and it was recommended to perform c‐myc, KDR, and VEGFR 2 molecular detection if necessary.

Integrating the patient's medical history, clinical manifestations, auxiliary examinations, and pathological results, we further diagnosed her with “left primary breast angiosarcoma” after a hospital‐wide multidisciplinary team (MDT) discussion. On March 23, 2020, the patient underwent “left residual breast resection + sentinel lymph node biopsy.” The operation was uneventful, and intraoperative frozen section pathology confirmed no residual tumor at the microscopic margin of the left breast tumor (R0 resection) and no metastasis in the sentinel lymph nodes.

On March 27, 2020, the postoperative pathological results were reported: (1) No residual cancer was found in the breast tissue around the surgical residual cavity of the left breast; (2) No cancer involvement was detected in the nipple, skin, or basal margin of the specimen; (3) No cancer metastasis was found in the left axillary sentinel lymph nodes (0/3) (Figure 2C,D). According to the 8th Edition AJCC Staging System [6], the final diagnosis was: left primary breast angiosarcoma (T1N0M0, Stage I). On April 3, 2020, the patient received the first cycle of postoperative adjuvant chemotherapy with the regimen of “doxorubicin + cyclophosphamide(4 cycles) followed by paclitaxel(4 cycles),” supplemented with symptomatic and supportive treatments such as gastric protection, liver protection, antiemetic, and immune enhancement. Blood routine and blood biochemistry were re‐examined within 1 week, showing no obvious abnormalities. The patient was discharged on April 10, 2020.

### 2.4 Follow‐Up

**FIGURE 3:**
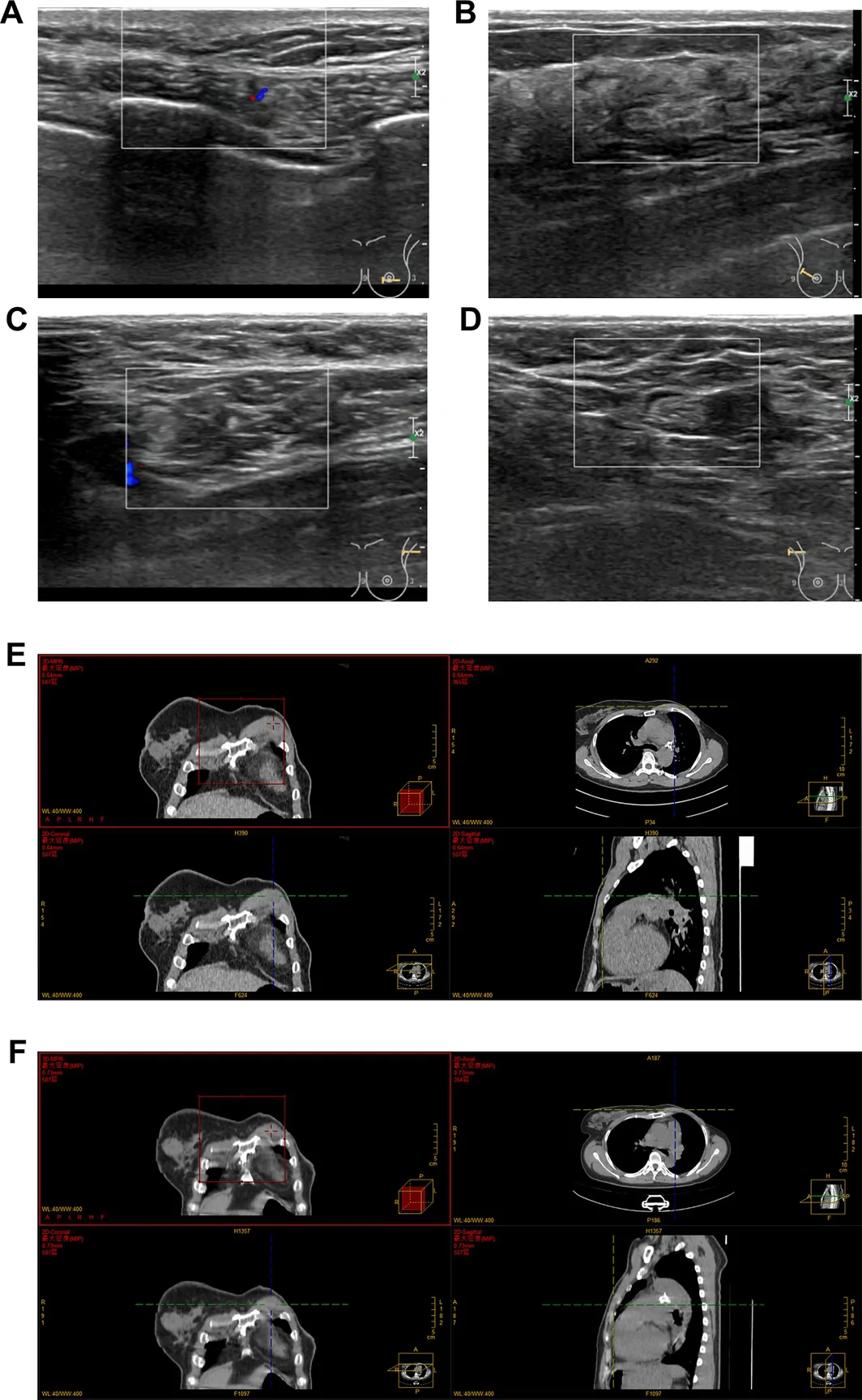
Postoperative follow‐up imaging examinations. (A–E) Follow‐up CDUS and chest CT images acquired on November 19, 2020, including left chest wall CDUS (A), right breast CDUS (B), bilateral axillary CDUS (C–D), and post‐mastectomy chest CT (E). (F) Post‐mastectomy multiplanar localized chest CT obtained on July 23, 2025.

After discharge, the patient received the 2nd to 8th cycles of postoperative adjuvant chemotherapy sequentially from April 24, 2020 to July 31, 2020. All chemotherapy courses were uneventful, and no obvious chemotherapy‐related adverse reactions occurred. The patient underwent re‐examination 1 month, 3 months, and 6 months after surgery, and then approximately every 6 months thereafter. The re‐examination items included breast CDUS, chest CT, CDUS of the digestive and urinary systems, blood routine, and tumor markers. Up to November 5, 2025 (follow‐up duration exceeding 5 years), no signs of local recurrence or distant metastasis were observed (Figure 3A–F).

Key clinical diagnosis and treatment milestones of this patient are summarized in Table 1.

**TABLE 1:** Timeline of the patient's diagnosis and treatment.

Time node	Treatment overview and outcomes
November 2019 (external hospital)	Imaging examination revealed a benign‐like left breast lesion (BI‐RADS 3), with regular follow‐up recommended.
March 2020 (initial visit)	The patient presented with rapidly enlarged breast mass. Imaging showed a left breast solid lesion (BI‐RADS 4a), while laboratory tests and tumor markers were normal. The lesion was preliminarily suspected to be benign fibroadenoma, and surgical resection was planned.
March 11, 2020	Primary breast mass resection was performed. Intraoperative frozen section indicated a vascular spindle cell tumor, and the operation was suspended for definite diagnosis.
March 17–20, 2020	Postoperative pathology combined with superior hospital consultation confirmed the diagnosis of low‐grade primary breast angiosarcoma.
March 23, 2020	Based on MDT discussion, completion breast resection and sentinel lymph node biopsy were performed, achieving R0 resection without lymph node metastasis.
March 27, 2020	Final pathology confirmed negative surgical margins and no tumor residue. The tumor was staged as stage I (T1N0M0).
April–July 2020	The patient completed 8 cycles of postoperative adjuvant chemotherapy uneventfully and was discharged with good recovery.
July 2020–November 2025	Long‐term follow‐up over 5 years showed no local recurrence or distant metastasis, with a favorable long‐term prognosis.

## 3 Discussion

Angiosarcoma is an aggressive malignant tumor of lymphatic or vascular endothelial origin with a propensity for early hematogenous metastasis [7], and is categorized etiologically into PAS and secondary angiosarcoma (SAS). It is a rare disease, and PAS occurring in the breast is even rarer than SAS. Due to its atypical clinical manifestations, PAS is often misdiagnosed in clinical practice. SAS usually develops secondarily in breast cancer patients who have received radiotherapy or complicated with chronic lymphedema after treatment (also known as Stewart‐Treves syndrome). It tends to involve the dermal and subcutaneous layers of the skin, not necessarily the parenchyma, with strong invasiveness and extremely poor prognosis [8]. The 5‐year overall survival (OS) rate is approximately 38%, and the recurrence‐free survival (RFS) period is about 43 months. Compared with SAS, PAS is generally not associated with a history of exposure to radioactive substances or chemical toxins, and the overall prognosis is slightly better than that of SAS, with a 5‐year OS rate of approximately 49% and an RFS period of about 44 months. The prognosis of PAS is often correlated with disease stage, tumor histological grade, and tumor size [9, 10].

The common clinical manifestations of PAS include breast masses with ill‐defined borders and rapid enlargement, which may be accompanied by or without skin changes. Physical examination can reveal breast masses that are cystic, cystic‐solid, or solid, with a smooth or irregular surface and variable mobility. Due to the atypical nature of its symptoms and signs, misdiagnosis is prone to occur; therefore, preoperative auxiliary differential examinations are particularly crucial. In the detection of routine breast malignant tumor markers (such as CEA, CA‐153, and CA‐125), PAS often presents negative results, indicating extremely low reference value. X‐ray imaging lacks specificity for the diagnosis of PAS, often showing masses or asymmetric shadows. If calcifications are present, they usually do not have the characteristics of malignant tumors. Due to the dense structure of breast glandular tissue, approximately one‐third of patients may have false‐negative mammography results [11]. On ultrasound imaging, PAS typically appears as heterogeneous hypoechoic, hyperechoic, or heterogeneous masses with irregular borders. CDUS can demonstrate increased blood flow signals, and cystic areas may be observed in the presence of hemorrhage or necrosis, but its specificity is also low [12]. Breast MRI is considered the most useful non‐invasive imaging modality for PAS diagnosis. It can detect heterogeneous signals within the mass, and contrast‐enhanced scanning can reveal abnormally high signals, indicating abundant blood flow inside the lesion [13]. Several studies have suggested that the combination of contrast‐enhanced ultrasound and MRI can improve the resolution of the enhanced morphology of breast angiosarcoma and better analyze hemodynamic characteristics, which is conducive to preoperative diagnosis [14].

Certainly, the gold standard for preoperative diagnosis of PAS still relies on pathological examinations, which include preoperative core needle biopsy, intraoperative frozen section, and postoperative routine pathological examinations. However, preoperative core needle biopsy often yields false‐negative results due to the high risk of bleeding and significant tumor heterogeneity. Therefore, the diagnosis of most PAS cases usually needs to be confirmed by postoperative pathology. Immunohistochemistry plays a crucial role in the differentiation and diagnosis of angiosarcoma [15]. CD31 and CD34 are sensitive markers for endothelial cells, and CD31, in particular, has high specificity and sensitivity. Factor VII‐related antigen (FVII) also serves as an endothelial cell marker, with a diagnostic positive rate of 40%–80%, and thus holds an important position in the diagnosis of PAS [16]. *Ulex europaeus* agglutinin I (UEA—1) is often regarded as an additional vascular endothelial marker with low specificity, which can be used for auxiliary diagnosis. ETS‐related gene (ERG) and Friend leukemia integration 1 transcription factor (FLI—1) are novel identification markers for breast angiosarcoma. Studies have shown that their sensitivity and specificity are higher than those of CD31 [17].

In this study, the patient was initially misdiagnosed with benign breast fibroadenoma preoperatively, largely due to our insufficient clinical recognition and vigilance toward rare PAS. Preoperative imaging showed inconsistent BI‐RADS grading, with ultrasonography indicating category 4A and mammography indicating category 3, which was predominantly suggestive of benign lesions. According to the ACR BI‐RADS 5th edition criteria, 2024 NCCN breast guidelines, and the 2021 CSBrS fibroadenoma guideline, preoperative contrast‐enhanced breast MRI and core needle biopsy are not routine or mandatory for such lesions [3, 4, 5]. Current guidelines recommend breast MRI only for high‐risk patients, radiologically indeterminate lesions with definite malignant suspicion, and preoperative staging of confirmed malignancies. Similarly, core needle biopsy is reserved for lesions with typical malignant imaging features, rather than discordant low‐grade BI‐RADS 4A lesions with coexisting benign mammographic findings. Therefore, these two examinations did not meet standardized clinical indications in this case. Nevertheless, the atypical preoperative presentation of PAS and our failure to conduct further targeted preoperative evaluation for the lesion's rapid growth feature led to preoperative misdiagnosis. Intraoperatively, when a rare malignant lesion was highly suspected, we promptly suspended the operation, awaiting definitive pathological diagnosis before formulating a subsequent standardized treatment protocol.

Complete surgical resection can significantly improve the survival rate of PAS [18]. The main surgical approaches include local resection, extended resection, and total mastectomy. Due to the strong invasiveness of PAS, which often invades the skin and underlying muscle tissue, resection involving the pectoralis major and minor muscles is sometimes selected. R0 resection of the tumor is the basis of surgical treatment, and total mastectomy is currently the most commonly used surgical method in clinical practice [19]. Furthermore, unlike conventional breast carcinoma that primarily spreads through lymphatic pathways, primary breast angiosarcoma is characterized by hematogenous metastasis as its dominant biological pattern. Although angiosarcoma may metastasize hematogenously to the liver, lungs, bone, brain and other organs at an early stage, regional lymph node invasion and metastasis are extremely rare; thus, routine axillary lymph node dissection is not recommended for this malignancy [20]. Considering the extremely low incidence of lymph node involvement and the unnecessary surgical trauma and postoperative complications caused by blind lymph node dissection, we only performed sentinel lymph node biopsy for accurate staging instead of extensive axillary dissection. In clinical practice, standardized intraoperative hemostasis and adhesive sealing techniques can effectively reduce postoperative seroma and bleeding complications after breast lymph node surgery, optimizing surgical safety and postoperative recovery [21, 22].

Postoperative adjuvant chemotherapy is considered to inhibit tumor recurrence and prolong OS, especially the regimen containing anthracyclines, ifosfamide, and paclitaxel, which has relatively good efficacy. Owing to the low incidence of this disease, the efficacy of chemotherapy has not been supported by large‐scale data studies, and only a small number of case reports or small‐sample studies have been reported [23, 24]. Radiotherapy is mainly recommended as local radiotherapy, especially for patients with tumor involvement of the skin and muscle tissue [9]. However, a study conducted by Skarentzos et al. [25] revealed that adjuvant chemotherapy significantly improves survival of patients with PAS (HR = 0.11, 95% CI: 0.02–0.45), while no survival benefit was observed with radiotherapy (*p* = 0.96). The rise of next‐generation sequencing (NGS) and research on immunotherapeutic targets have brought targeted therapy and immunotherapy into public view. Painter et al. [26] proposed through a genomic project study that phosphatidylinositol‐3‐kinase catalytic alpha subunit (PIK3C‐α) inhibitors are expected to become new therapeutic targets for breast angiosarcoma. With the deepening understanding and increasing attention to this disease, it is believed that the treatment methods for PAS will be more improved in the future.

In this case, postoperative immunohistochemistry showed positive vascular markers (CD31, CD34, FLT‐1, Vimentin) and negative epithelial markers (EMA, CKpan), confirming vascular endothelial origin of the tumor. Combined with typical angiosarcoma morphological features, the final diagnosis was low‐grade primary breast angiosarcoma (T1N0M0, stage I). Ki‐67 is an independent prognostic biomarker for angiosarcoma, and the 25% index implies moderate proliferation with inherent recurrence risk in early‐stage lesions [17]. Considering the tumor's malignant potential, we performed total mastectomy plus sentinel lymph node biopsy for radical resection, followed by adjuvant chemotherapy to reduce postoperative recurrence. Based on the established histopathological diagnosis of low‐grade breast angiosarcoma, we did not pursue further molecular testing for c‐myc, KDR, or VEGFR2, as these markers are primarily utilized for differential diagnosis in equivocal cases or for distinguishing primary from secondary angiosarcoma rather than providing additional clinically actionable information in this context.

Previous studies have reported that postmenopausal breast angiosarcoma cases are predominantly secondary angiosarcoma following radiation therapy, typically presenting with large tumors and typical malignant imaging features [27, 28]. In contrast, this postmenopausal patient presented with a primary breast angiosarcoma manifesting as a small, well‐circumscribed, rapidly growing mass with contradictory BI‐RADS grades, mimicking benign fibroadenoma and causing guideline‐conformant preoperative underestimation. This case innovatively summarizes the misdiagnosis pitfalls of atypical low‐grade PAS in postmenopausal women, verifies the value of intraoperative surgical suspension and superior pathological consultation, and supplements long‐term survival data of early PAS with moderate Ki‐67 activity. Despite increased patient burden from staged surgery, individualized treatment and multidisciplinary collaboration achieved over 5‐year recurrence‐free survival. This study enriches the clinical and prognostic data of postmenopausal PAS. Clinicians should enhance PAS recognition, vigilantly evaluate rapidly growing benign‐appearing breast masses, and adopt standardized multimodal and multidisciplinary management to reduce misdiagnosis.

## 4 Conclusion

This case reports an atypical low‐grade postmenopausal PAS mimicking fibroadenoma, which is easily misdiagnosed under routine guidelines. Intraoperative conditional surgery suspension and pathological consultation ensured accurate diagnosis. Individualized surgery combined with adjuvant chemotherapy achieved favorable long‐term survival. This study supplements the prognostic data of early atypical PAS and reminds clinicians to raise vigilance toward rapidly growing benign‐like breast masses for precise multidisciplinary management of rare breast sarcomas.

## Author Contributions

**Qingwei Wang:** methodology, validation, resources, writing – review and editing, supervision. **Zeyi Ye:** supervision, validation, writing – review and editing, resources. **Changan Yang:** conceptualization, writing – original draft, writing – review and editing, project administration, resources. **Zhiqiang Zheng:** investigation, validation, writing – review and editing. **Tianzi Hong:** conceptualization, writing – original draft, writing – review and editing, supervision, resources. **Tingting Liu:** conceptualization, funding acquisition, project administration, writing – review and editing, writing – original draft, formal analysis. **Hongming Lin:** methodology, data curation, writing – review and editing, visualization, software. **Shiya Yu:** data curation, investigation, software, visualization, writing – review and editing. **Yueqin Xu:** formal analysis, writing – review and editing, conceptualization.

## Funding

This work was supported by the Quanzhou Science and Technology Program Project, Fujian, China (No. 2025QZNYZ049).

## Disclosure

During the preparation of this work the authors did not use generative AI or AI‐assisted technologies to create or interpret any core content.

## Ethics Statement

This case report was conducted based on a patient enrolled in our approved institutional research project. Written informed consent was obtained from the patient and their family prior to manuscript writing and publication, including consent for the publication of all relevant clinical data and accompanying images (no. 20251119). This study was reviewed and approved by the Ethics Committee of Jinjiang Municipal Hospital (Shanghai Sixth People‘s Hospital Fujian) (no. jjsyyll‐2026‐064). All procedures complied with the ethical standards of the institutional research committee, and no ethical conflict exists in this case report.

## Conflicts of Interest

The authors declare no conflicts of interest.

## Data Availability

The data that support the findings of this study are available on request from the corresponding author. The data are not publicly available due to privacy or ethical restrictions.
